# Syntenin promotes VEGF-induced VEGFR2 endocytosis and angiogenesis by increasing ephrin-B2 function in endothelial cells

**DOI:** 10.18632/oncotarget.16452

**Published:** 2017-03-22

**Authors:** Nara Tae, Suhyun Lee, Okwha Kim, Juhee Park, Sunghun Na, Jeong-Hyung Lee

**Affiliations:** ^1^ Department of Biochemistry, College of Natural Sciences, Kangwon National University, Chuncheon, Gangwon-Do 243-41, Republic of Korea; ^2^ Department of Obstetrics and Gynecology, Kangwon National University Hospital, school of Medicine, Kangwon National University, Chuncheon, Gangwon-Do 243-41, Republic of Korea

**Keywords:** syntenin, VEGFR2 endocytosis, ephrin-B2, endothelial cell, angiogenesis

## Abstract

Syntenin, a tandem PDZ-domain-containing scaffold protein, is involved in the regulation of diverse biological functions, including protein trafficking, exosome biogenesis, and cancer metastasis. Here, we present the first study to explore the significance of syntenin in endothelial cells. Syntenin knockdown in human umbilical vein endothelial cells (HUVECs) impaired vascular endothelial growth factor (VEGF)-mediated proliferation, migration, invasion, vascular permeability, and nitric oxide (NO) production. Syntenin knockdown also suppressed expression of the VEGFR2 target genes *VEGF, MMP2*, and *Nurr77* as well as VEGF-induced angiogenesis *in vitro* and *in vivo*. And it decreased cell-surface levels of ephrin-B2. Biochemical analyses revealed that syntenin exists in complex with VEGFR2 and ephrin-B2. Syntenin knockdown abolished the association between VEGFR2 and ephrin-B2, suggesting syntenin functions as a scaffold protein facilitating their association in HUVECs. Consistent with these observations, knocking down syntenin or ephrin-B2 abolished VEGF-induced endocytosis and VEGFR2 phosphorylation and activation of its downstream signaling molecules. Treatment with MG132, a proteasome inhibitor, rescued the downregulation of ephrin-B2 and VEGFR2 signaling induced by syntenin knockdown. These findings demonstrate that syntenin promotes VEGF signaling and, through its PDZ-dependent interaction with ephrin-B2, enhances VEGF-mediated VEGFR2 endocytosis and subsequent downstream signaling and angiogenesis in endothelial cells.

## INTRODUCTION

Angiogenesis is a complex, multi-step process, which involves destabilization of the established vessel, followed by EC proliferation, migration, and tubulogenesis [1, 2]. VEGF is the crucial regulator of angiogenesis [1], acting on ECs as both a chemotactic and a mitogenic factor via endothelial cell-specific receptors: VEGFR1 (Flt-1), VEGFR2 (Flk-1/KDR), and VEGFR3 (Flt-4). VEGFR2 is considered to be the major transducer of VEGF-mediated effects on ECs, such as induction of proliferation, survival, migration, and permeability [1, 2]. In ECs, VEGFR2 signaling activates numerous downstream signaling mediators, including phosphoinositide-3 kinase (PI3K)/AKT, PLCγ, p38 mitogen-activated protein kinase, and extracellular-signal-regulated kinase-1/2 (ERK-1/2), which act in a coordinated manner to initiate the angiogenic process [3]. The PI3K/AKT pathway is responsible for the production of NO, an essential mediator of VEGF-stimulated endothelial permeability and postnatal angiogenesis, through the activation of eNOS in ECs [4–6].

VEGFR2 was thought to signal from the plasma cell membrane after ligand-induced dimerization and activation [7, 8]. However, a number of recent studies have demonstrated the importance of endocytosis and trafficking in regulation of VEGFR2 signaling and angiogenesis in ECs [9, 10]. After ligand binding, VEGFR2 is endocytosed via a classical clathrin-mediated pathway and targeted either for recycling back to the plasma membrane or for sequential proteasomal and lysosomal degradation. Cargo movement from clathrin-coated vesicles to Rab5 endosomes is thought to be controlled by the adaptor proteins APPL1 and APPL2 [11]. Upon entry into the cell, VEGFR2 continues to signal. VEGFR2 endocytosis is regulated by several transmembrane and cytosolic interacting proteins [10]. Ephrin-B2, a transmembrane protein belonging to the B-class ephrin subfamily, and its intracellular interactors, clathrin-associated protein disabled 2 (Dab2) and cell polarity regulator PAR-3, promote clathrin-dependent VEGFR internalization and thereby downstream signaling and angiogenesis [12, 13]. Disruption of this interaction by silencing of Dab2 or PAR-3 reduces VEGFR2 internalization and impairs VEGF-induced angiogenesis [12]. Deletion of ephrin-B2 eliminates the constitutive and VEGF-induced internalization of VEGFR2 and profoundly impairs its signaling [13]. Ephrin-B2 has a C-terminal PDZ (PSD-95/Dlg/ZO-1)-binding motif for the binding of PDZ domain containing proteins [14]. Mutation of this C-terminal PDZ-binding motif impairs endocytosis of VEGFR2, which reduces sprouting angiogenesis under physiological and pathological conditions, and VEGFR2 downstream signaling [13, 15]. Although the mechanism by which ephrin-B2 promotes the initiation of VEGFR2 endocytosis is unclear, the interaction of ephrin-B2 with its PDZ domain effectors through the C-terminal PDZ binding motif may be required for its regulation of VEGFR2 endocytosis, signaling, and angiogenesis.

Syntenin (also known as syndecan-binding protein, SDCBP) was initially cloned from terminally differentiating human melanoma cells as melanoma differentiation-associated gene-9 (MDA-9) [16, 17]. Syntenin is a 32 kDa protein that comprises a 113-amino-acid N-terminal domain with no obvious structural motifs, followed by two adjacent tandem PDZ domains and a short 24-amino-acid C-terminal domain [18]. The PDZ domains of syntenin are essential for assembly and organization of diverse cell signaling processes that occur at the plasma membrane [19, 20]. The PDZ domains bind to phosphatidylinositol 4,5-bisphosphate (PIP2) with high affinity [21] and multiple peptide motifs with low-to-medium affinity [22, 23]. This plasticity allows syntenin to participate in multiple biological functions, including receptor clustering, protein trafficking, exosome biogenesis, and transcription factor activation [19, 20, 24–26]. Syntenin is highly expressed in several cancer cell types and tissues and regulates tumor cell invasion and metastasis [19, 20, 26–33]. Syntenin also regulates tumor angiogenesis by inducing the expression of angiogenic factors such as interleukin-8 and growth factor-binding protein-2 (IGFBP-2) in melanoma cells [34]. In the present study, we used siRNA and shRNA knockdown to study the role of syntenin in VEGF-mediated EC migration, proliferation, and invasion, and *in vitro* and *in vivo* angiogenesis. This work provides the first study of the significance of syntenin in ECs to date.

## RESULTS

### Downregulation of syntenin inhibits VEGF-induced proliferation, migration, and invasion of HUVECs

**Figure 1:**
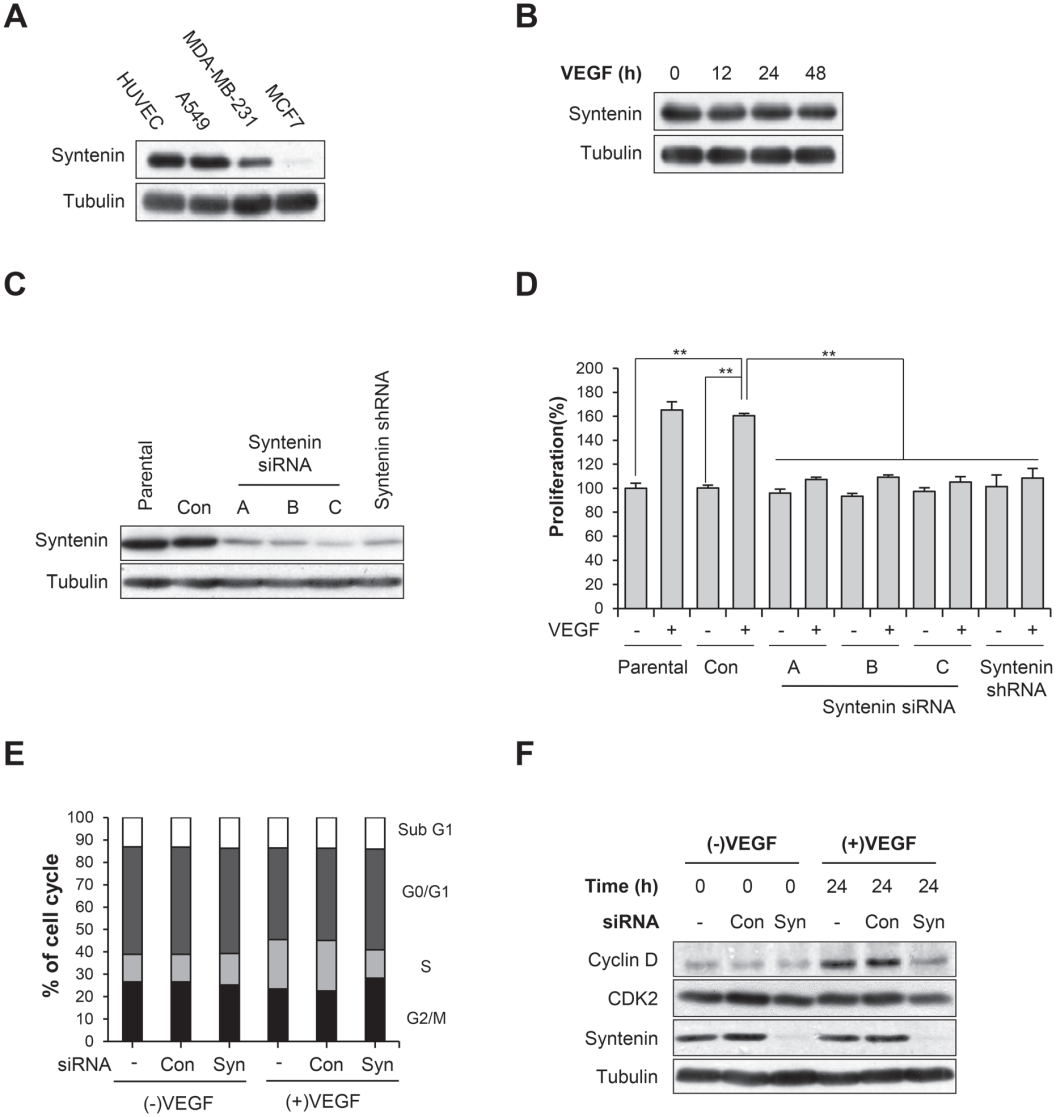
Syntenin is required for VEGF-induced proliferation of HUVECs **(A)** HUVECs express syntenin. Whole cell lysates were prepared from the indicated cells and then blotted with the indicated antibodies. **(B)** Serum-starved HUVECs were stimulated with VEGF (40 ng/mL) for indicated periods of time. Whole cell lysates were blotted with the indicated antibodies. **(C)** HUVECs were transfected with control siRNA (Con) or the indicated syntenin siRNAs or infected with lentiviral syntenin shRNA. Whole cell lysates were blotted with the indicated antibodies. **(D)** HUVECs transfected with control siRNA (Con) or the indicated syntenin siRNAs, or infected with lentiviral syntenin shRNA were incubated with or without VEGF (40 ng/mL) for 48 h. Cell proliferation was determined by BrdU proliferation assay. *Columns*, mean of three independent experiments performed in triplicate; *bars*, S.D.; **, *P*<0.01. **(E)** HUVECs transfected with control siRNA (Con) or syntenin siRNA (Syn) were incubated with or without VEGF (40 ng/mL) for 24 h, and percent distribution of cells in each stage of the cell cycle was analyzed by flow cytometry with PI staining. **(F)** HUVECs transfected with control siRNA (Con) or syntenin siRNA (Syn) were incubated with or without VEGF (40 ng/mL) for 24 h. Whole cell lysates were blotted with the indicated antibodies.

**Figure 2:**
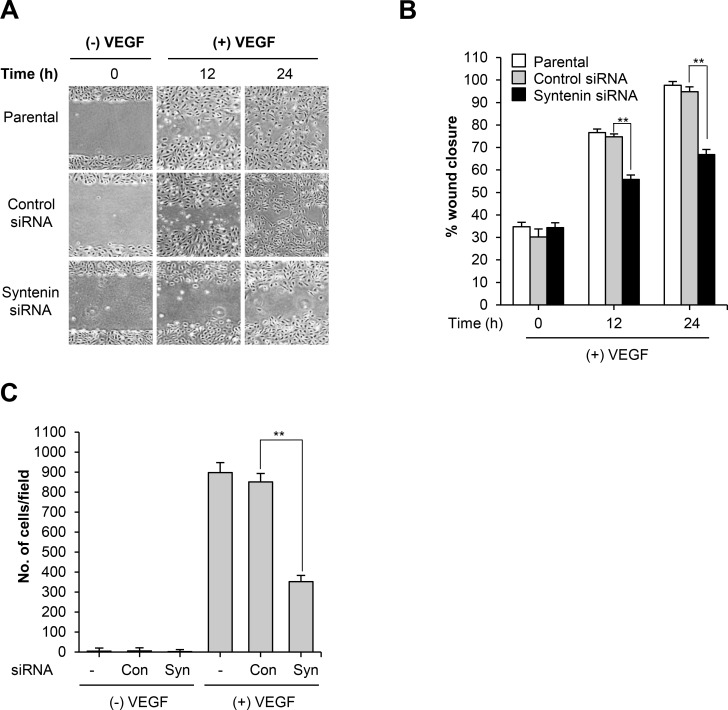
Knockdown of syntenin inhibits VEGF-induced migration and invasion **(A)** HUVECs transfected with control siRNA (Con) or syntenin siRNA (Syn) underwent a wound-healing assay in the presence of VEGF (40 ng/mL) for the indicated periods of time. Representative images are shown. **(B)** The graph represents the progression of wound healing at indicated time points. *Columns*, mean of two independent experiments performed in triplicate; *bars*, S.D.; **, *P*<0.01. **(C)** HUVECs transfected with control siRNA (Con) or syntenin siRNA (Syn) underwent an invasion assay in the presence or absence of VEGF (40 ng/mL). *Columns*, mean of two independent experiments performed in triplicate; *bars*, S.D.; **, *P*<0.01.

We first determined that syntenin was abundantly expressed in HUVECs (Figure 1A), comparable to the level in A549 human adenocarcinoma cells, and that stimulation with VEGF did not alter the level of syntenin (Figure 1B). We next examined whether syntenin regulates VEGF-induced EC responses. To knock down syntenin expression in HUVECs, we tested syntenin silencing efficiency using three different syntenin siRNAs and a syntenin shRNA. All effectively downregulated the expression level of syntenin (Figure 1C) and inhibited VEGF-induced proliferation of HUVECs, as assessed by MTT and BrdU incorporation assays (Supplementary Figure 1 and Figure 1D), as well as cell migration (Supplementary Figure 2) to a similar extent. We selected syntenin siRNA-C for further experiments. Cell cycle analyses also revealed that syntenin knockdown inhibited VEGF-induced cell cycle progression of HUVECs (Figure 1E). Consistent with these observations, syntenin knockdown significantly impaired VEGF-induced cyclin D1 expression in HUVECs (Figure 1F). We next performed a wound-healing migration assay on HUVEC monolayers transfected with control siRNA or syntenin siRNA. Knockdown of syntenin by siRNA significantly suppressed VEGF-induced wound closure by HUVECs, compared to HUVEC monolayers transfected with control siRNA (Figure 2A and 2B). Moreover, syntenin siRNA knockdown significantly suppressed VEGF-induced invasion of HUVECs through Matrigel (Figure 2C).

### Downregulation of syntenin inhibits VEGF-induced angiogenesis and NO production

**Figure 3:**
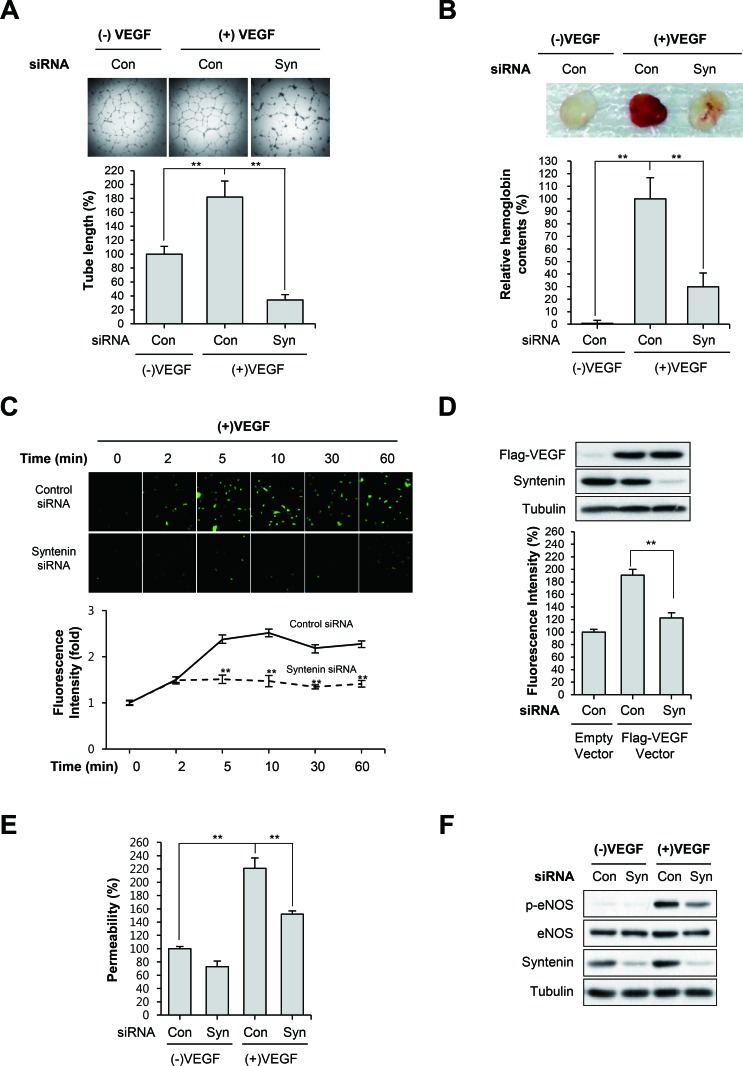
Knockdown of syntenin inhibits VEGF-induced vascular permeability and angiogenesis **(A)** HUVECs transfected with control siRNA (Con) or syntenin siRNA (Syn) were subjected to a tube formation assay in the presence or absence of VEGF (40 ng/mL) for 18 h. Representative images are shown (top). The graphs represent the length of tube in the capillary networks (bottom). *Columns*, mean of three independent experiments performed in duplicate; *bars*, S.D.; **, *P*<0.01. **(B)** C57BL/6 mice were implanted subcutaneously with Matrigel plugs containing control siRNA (Con) or syntenin siRNA (Syn) with or without VEGF. After 14 days, Matrigel plugs were excised. Representative photos are shown (top). Excised matrigel plugs were homogenized and the hemoglobin level was determined by Drabkin Reagent (bottom). *Columns*, mean of two independent experiments performed in triplicate; *bars*, S.D.; **, *P*<0.01. **(C)** Effect of syntenin downregulation on VEGF-induced NO production. HUVECs transfected with control siRNA (Con) or syntenin siRNA (Syn) were incubated with VEGF (40 ng/mL) for the indicated periods of time. NO production was determined by DAF2-DA. Representative images are shown (top). The graphs represent the fluorescence intensity at the indicated time points (bottom). *Columns*, mean of two independent experiments performed in triplicate; *bars*, S.D.; **, *P*<0.01. **(D)** A Flag-tagged VEGF expression vector was introduced into control siRNA (Con)- or syntenin siRNA (Syn)-transfected HUVECs. NO production was determined by DAF2-DA. This is a representative Western blot for Flag-VEGF and syntenin from the lysates. **(E)** HUVECs transfected with control siRNA (Con) or syntenin siRNA (Syn) underwent a permeability assay with or without VEGF (40 ng/mL). *Columns*, mean of three independent experiments performed in triplicate; *bars*, S.D.; **, *P*<0.01. **(F)** HUVECs transfected with control siRNA (Con) or syntenin siRNA (Syn) were incubated with or without VEGF (40 ng/mL) for 10 min. Whole cell lysates were blotted with the indicated antibodies.

Matrigel tube formation assays revealed that syntenin knockdown significantly impaired VEGF-induced capillary-like tube formation by HUVECs (Figure 3A). We next evaluated the impact of syntenin downregulation on *in vivo* angiogenesis. Matrigel plugs containing control siRNA or mouse syntenin siRNA were implanted subcutaneously into mice, and vessel formation in response to VEGF was determined by measuring hemoglobin levels in the plugs. Vessel formation in control siRNA-containing Matrigel plugs was increased in response to VEGF. In syntenin siRNA plugs, VEGF-induced vessel formation was significantly reduced compared with control siRNA-containing implants (Figure 3B).

NO production plays a prominent role in VEGF-induced angiogenesis and vascular permeability [4, 5]. Thus, we assessed the effect of syntenin knockdown on VEGF-induced NO production and permeability of EC monolayers. We measured intracellular NO levels by loading cells with the NO-sensitive fluorescent probe DAF2-DA. VEGF stimulation of control siRNA-transfected HUVECs increased NO production in a time-dependent manner; however, HUVECs transfected with syntenin siRNA exhibited significantly impaired VEGF-induced NO production (Figure 3C). Overexpression of VEGF in syntenin knockdown cells also decreased the level of NO (Figure 3D). The ability of VEGF to increase the passage of FITC-labeled dextran across a monolayer of HUVECs was also blocked when HUVECs were transfected with syntenin siRNA (Figure 3E). Interestingly, knockdown of syntenin with siRNA significantly suppressed VEGF-induced eNOS phosphorylation on Ser1177 (Figure 3F), which plays a critical role in eNOS activation [35]. Taken together, these results demonstrate that downregulation of syntenin inhibits VEGF-stimulated angiogenesis and endothelial processes, such as NO production and permeability.

### Downregulation of syntenin inhibits VEGFR2 signaling and the expression of its target genes

**Figure 4:**
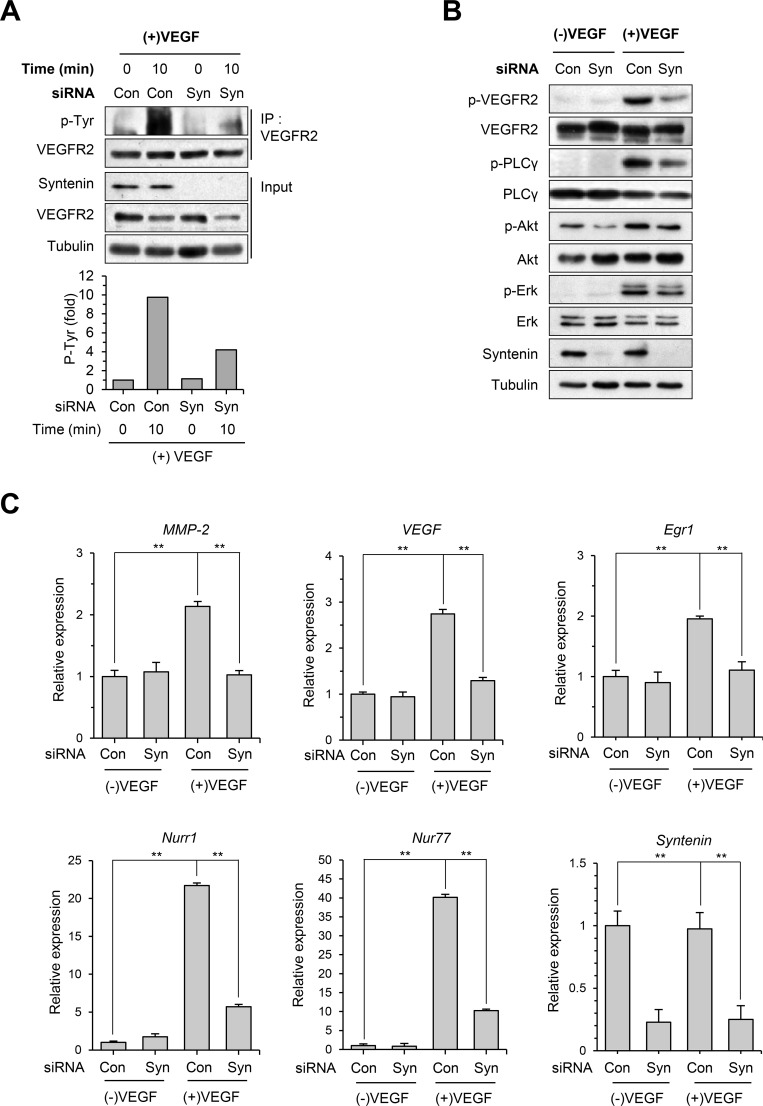
Knockdown of syntenin inhibits VEGF-induced activation of VEGFR2 and its downstream molecules **(A)** HUVECs transfected with control siRNA (Con) or syntenin siRNA (Syn) were incubated with or without VEGF (40 ng/mL) for 10 min. Whole cell lysates were immunoprecipitated (IP) with VEGFR2 antibody. The immunoprecipitates and input were blotted with the indicated antibodies. The graphs represent quantification of the levels of VEGFR2 p-Tyr normalized to VEGFR2. **(B)** HUVECs transfected with control siRNA (Con) or syntenin siRNA (Syn) were incubated with or without VEGF (40 ng/mL) for 10 min. Whole-cell lysates were blotted with the indicated antibodies. **(C)** HUVECs transfected with control siRNA (Con) or syntenin siRNA (Syn) were incubated with or without VEGF (40 ng/mL) for 45 min. Total RNAs were harvested and subjected to real-time qPCR. *Columns*, mean of two independent experiments performed in triplicate; *bars*, S.D.; **, *P*<0.01.

To investigate the mechanisms by which syntenin regulates VEGF-induced angiogenesis and endothelial processes, we next examined whether downregulation of syntenin affects VEGF-induced activation of signaling molecules. Knockdown of syntenin by siRNA significantly decreased the phosphorylation level of VEGFR2 on Tyr residues and specifically on Tyr 1175 in HUVECs (Figure 4A and 4B). Consistent with these observations, syntenin knockdown significantly suppressed VEGF-induced phosphorylation of VEGFR2 downstream signaling molecules, such as PLCγ, AKT, and ERK. Real-time qPCR confirmed that syntenin knockdown significantly suppressed VEGF-induced expression of *MMP-2, VEGF, Egr1, Nurr1*, and *Nur77* (Figure 4C). These results demonstrate that syntenin may be necessary for VEGF-induced VEGFR2 activation and thereby downstream signaling.

### Syntenin associates with VEGFR2 and ephrin-B2, and knockdown of syntenin disrupts this association

**Figure 5:**
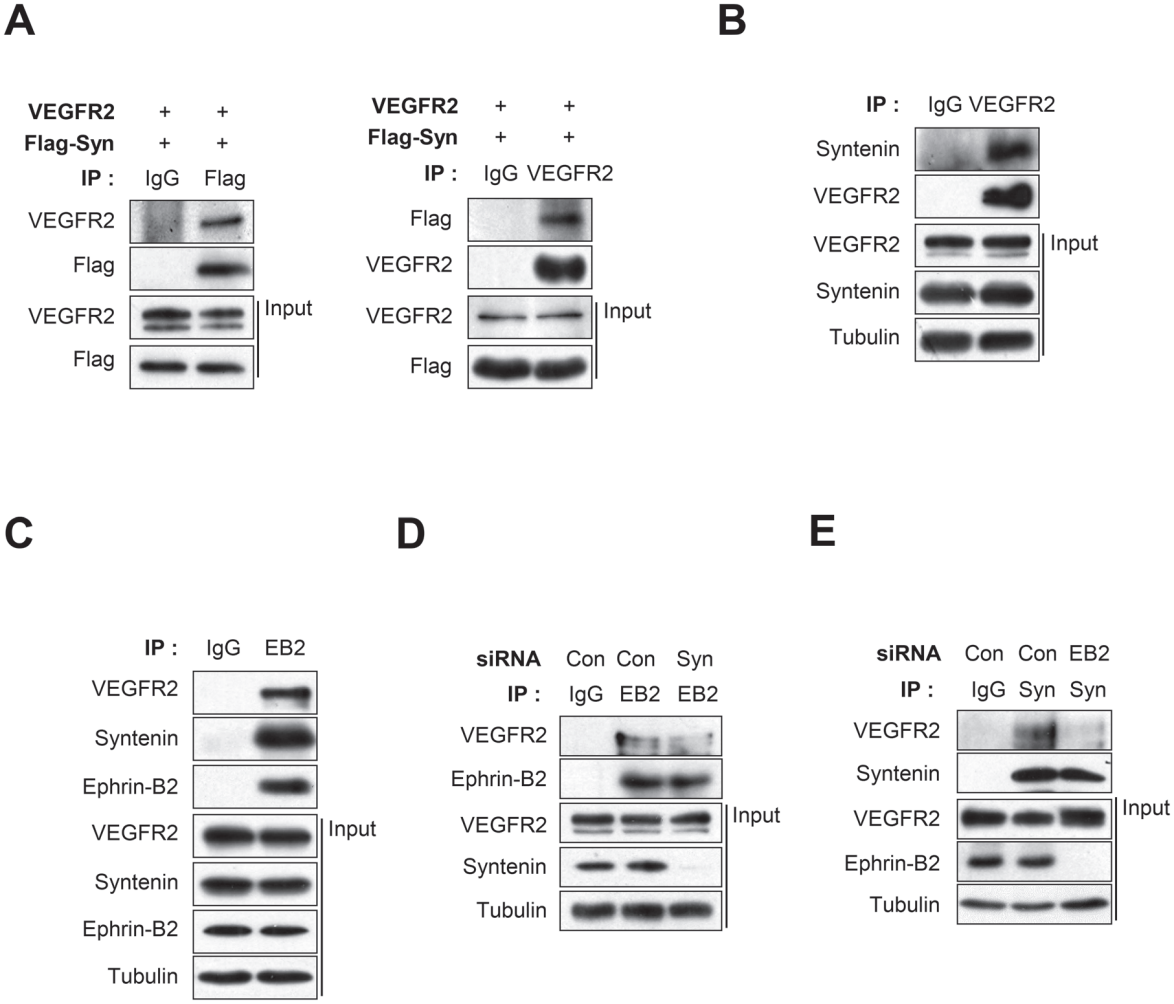
Syntenin associates with both VEGFR2 and ephrin-B2, and knockdown of syntenin disrupts their association **(A)** HEK293 cells were transfected with VEGFR2 and Flag-syntenin. Whole cell lysates were immunoprecipitated (IP) with Flag or VEGFR2 antibody. The immunoprecipitates and input were blotted with the indicated antibodies. **(B)** Whole cell lysates from HUVECs were immunoprecipitated (IP) with VEGFR2 antibody. The immunoprecipitates and input were blotted with the indicated antibodies. **(C)** Whole cell lysates from HUVECs were immunoprecipitated (IP) with ephrin-B2 antibody (EB2). The immunoprecipitates and input were probed with the indicated antibodies. **(D)** HUVECs were transfected with control siRNA (Con) or syntenin siRNA (Syn). Whole cell lysates were immunoprecipitated (IP) with ephrin-B2 antibody (EB2). The immunoprecipitates and input were probed with the indicated antibodies. **(E)** HUVECs were transfected with control siRNA (Con) or ephrin-B2 siRNA (EB2). Whole cell lysates were immunoprecipitated (IP) with syntenin antibody (Syn). The immunoprecipitates and input were blotted with the indicated antibodies.

Co-immunoprecipitation revealed that syntenin was associated with VEGFR2 in HEK293 cells (Figure 5A) and HUVECs (Figure 5B). Syntenin associates with ephrin-B2 [23, 36]. Ephrin-B2 associates with VEGFR2 and regulates VEGF-induced angiogenesis and VEGFR2 endocytosis through PDZ interactions at its C-terminal PDZ-binding motif in endothelial cells [13]. Thus, we hypothesized that syntenin may regulate the association of ephrin-B2 with VEGFR2 in endothelial cells. To test this hypothesis, we used co-immunoprecipitation to determine the effect of syntenin on the association of VEGFR2 with ephrin-B2 in HUVECs. Syntenin and VEGFR2 were detected in ephrin-B2 immunoprecipitates (Figure 5C), suggesting that syntenin forms a complex with VEGFR2 and ephrin-B2 in HUVECs. Interestingly, syntenin siRNA knockdown significantly impaired the association of VEGFR2 with ephrin-B2 (Figure 5D). Knockdown of ephrin-B2 by siRNA also impaired the association of syntenin with VEGFR2 (Figure 5E). These results demonstrate that the tandem PDZ protein syntenin may increase the association of VEGFR2 with ephrin-B2, which is critical for VEGF-induced VEGFR2 endocytosis and angiogenesis [13].

### Downregulation of syntenin blocks VEGF-induced VEGFR2 endocytosis in HUVECs

**Figure 6:**
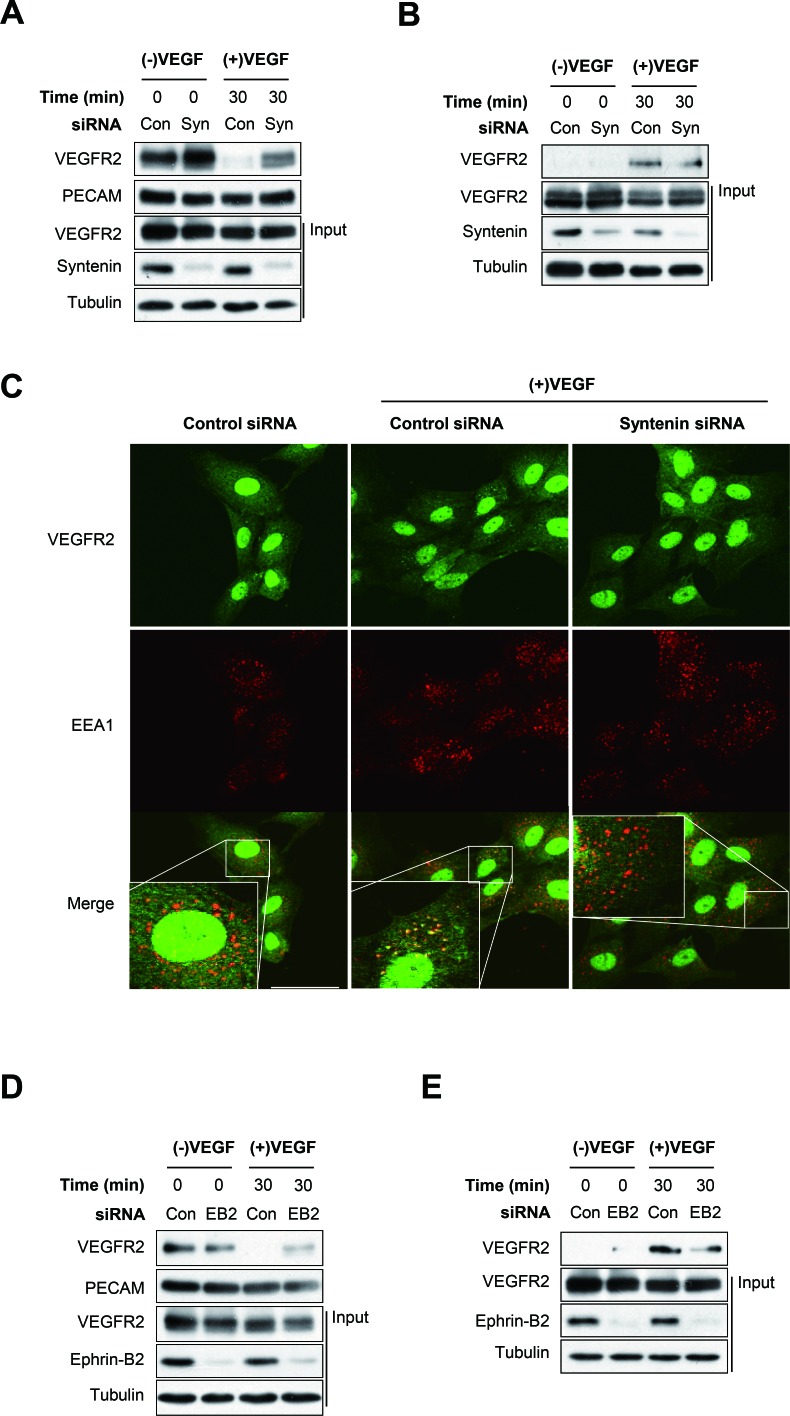
Syntenin regulates VEGF-induced VEGFR2 internalization **(A)** HUVECs transfected with control siRNA (Con) or syntenin siRNA (Syn) were incubated with VEGF (40 ng/mL) for the indicated periods of time and then labeled with biotin for 30 min. Whole cell lysates were subjected to streptavidin pull-down and then blotted with the indicated antibodies. **(B)** HUVECs transfected with control siRNA (Con) or syntenin siRNA (Syn) were surface labeled with biotin and then incubated with VEGF (40 ng/mL) for the indicated periods of time. After stripping the remaining cell-surface biotin, whole cell lysates were prepared and subjected to streptavidin pull-down and then were blotted with the indicated antibodies. **(C)** HUVECs transfected with control siRNA (Con) or syntenin siRNA (Syn) were incubated with VEGF (40 ng/mL) for 15 min, and then immunostained with VEGFR2 or EEA1 antibody. Representative images of immunostaining results are shown. Bar = 50 μm. **(D)** HUVECs transfected with control siRNA (Con) or ephrin-B2 siRNA (EB2) were stimulated with VEGF (40 ng/mL) for the indicated periods of time and then labeled with biotin for 30 min. Whole cell lysates were subjected to streptavidin pull-down and then blotted with the indicated antibodies. **(E)** HUVECs transfected with control siRNA (Con) or ephrin-B2 siRNA (EB2) were surface labeled with biotin and then stimulated with VEGF (40 ng/mL) for the indicated periods of time. After stripping the remaining cell-surface biotin, whole cell lysates were prepared and subjected to streptavidin pull-down and were blotted with the indicated antibodies.

We next investigated the effect of syntenin on VEGF-induced VEGFR2 endocytosis in endothelial cells. Knockdown of syntenin by siRNA significantly inhibited the VEGF-induced decrease in VEGFR2 expression at the plasma membrane, as assessed by surface biotinylation assay (Figure 6A). Consistently, siRNA knockdown of syntenin significantly inhibited the VEGF-induced increase in VEGFR2 internalization, as determined by biotin internalization assay (Figure 6B). Immunofluorescence imaging confirmed that knockdown of syntenin significantly decreased VEGF-induced colocalization of VEGFR2 with the endosome marker EEA1 (Figure 6C). Immunofluorescence also revealed that downregulation of syntenin decreased the VEGF-induced colocalization of phospho-VEGFR2 with EEA1 (Supplementary Figure 3). Similar to the results of syntenin downregulation, siRNA knockdown of ephrin-B2 inhibited the VEGF-induced decrease in VEGFR2 expression at the plasma membrane and the increase in VEGFR2 internalization (Figure 6D and 6E, Supplementary Figure 4). Taken together, these results demonstrate that syntenin could be necessary for VEGF-induced VEGFR2 endocytosis by enhancing the association of VEGFR2 with ephrin-B2.

### Downregulation of syntenin induces degradation of ephrin-B2 in HUVECs

**Figure 7:**
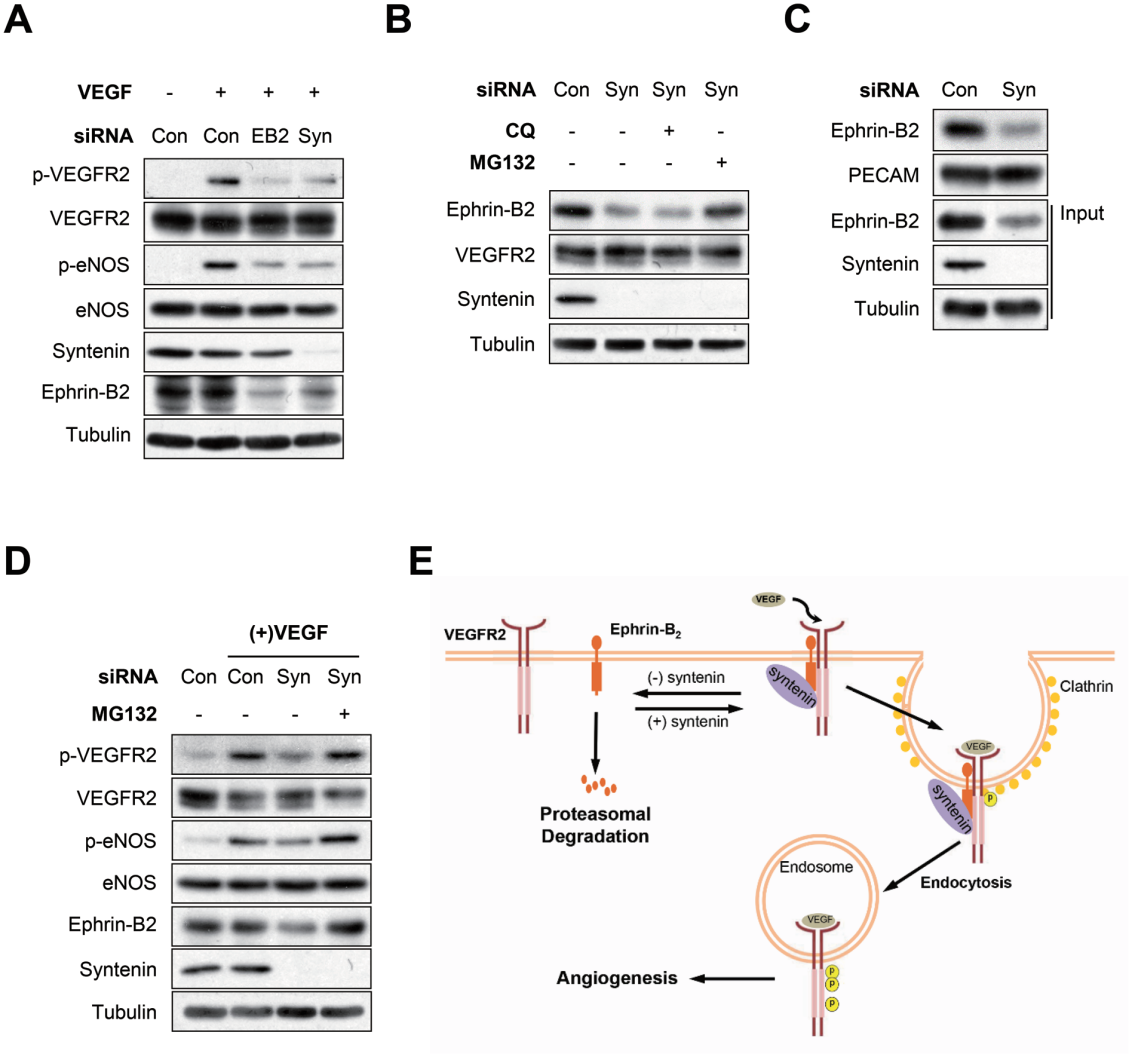
Syntenin regulates ephrin-B2 expression at the plasma membrane **(A)** Effect of syntenin or ephrin-B2 downregulation on VEGF-induced VEGFR2 phosphorylation. HUVECs transfected with control siRNA, syntenin siRNA, or ephrin-B2 siRNA were incubated with VEGF (40 ng/mL) for 10 min. Whole cell lysates were blotted with the indicated antibodies. **(B)** HUVECs transfected with control siRNA or syntenin siRNA were treated with chloroquine (CQ, 100 μM) or MG132 (10 μM) for 6 h. Whole cell lysates were blotted with the indicated antibodies. **(C)** HUVECs transfected with control siRNA (Con) or syntenin siRNA (Syn) were labeled with biotin for 30 min. Whole cell lysates were subjected to streptavidin pull-down and then blotted with the indicated antibodies. **(D)** Effect of MG132 on syntenin-mediated VEGF signaling. HUVECs transfected with control siRNA or syntenin siRNA were treated with MG132 (10 μM) for 6 h and then incubated with VEGF (40 ng/mL) for 10 min. Whole cell lysates were blotted with the indicated antibodies. **(E)** Working model of syntenin in regulating VEGF-induced VEGFR2 endocytosis and angiogenesis.

We next asked whether downregulation of syntenin impaired VEGF-induced phosphorylation of VEGFR2 and eNOS in HUVECs, in a manner similar to downregulation of ephrin-B2 (Figure 7A). Interestingly, syntenin knockdown significantly decreased the expression level of ephrin-B2 in HUVECs. Treatment with MG132, a proteasome inhibitor, blocked the downregulation of ephrin-B2 induced by syntenin siRNA; however, the lysosome inhibitor chloroquine had no such effect (Figure 7B). A surface biotinylation assay also revealed that syntenin knockdown decreased the expression level of ephrin-B2 at the cell surface (Figure 7C). Treatment of HUVECs with MG132 rescued the reduction in VEGFR2 signaling induced by downregulation of syntenin (Figure 7D). These results suggested that syntenin increases cell surface expression of ephrin-B2 by preventing proteasome-mediated degradation in ECs, and thereby VEGF-mediated VEGFR2 endocytosis and downstream signaling.

## DISCUSSION

VEGFR2 is one of many receptor tyrosine kinases that undergo endocytosis in response to ligand stimulation. After VEGF stimulation, VEGFR2 is internalized within clathrin-coated vesicles [11]. The endocytosis of VEGFR2 plays a critical role in VEGF-induced angiogenesis and its signaling [12, 13]. In the current study, we showed that syntenin, a tandem PDZ-domain protein that induces tumor cell invasion and metastasis, functions as a positive regulator of VEGF-induced angiogenesis by enhancing VEGF-induced VEGFR2 endocytosis and subsequent activation of VEGFR2 signaling molecules in ECs (Figure 7E). Syntenin knockdown in ECs decreased VEGF-induced proliferation, migration, invasion, NO production, and angiogenesis. Importantly, syntenin co-immunoprecipitated with VEGFR2 and ephrin-B2, and syntenin knockdown disrupted the association of VEGFR2 with ephrin-B2 and VEGF-induced internalization of VEGFR2, as did ephrin-B2 knockdown, resulting in suppression of the VEGF-induced activation of VEGFR2 and its downstream signaling molecules such as AKT, eNOS, and PLCγ. Moreover, knockdown of syntenin decreased the cell surface expression level of ephrin-B2. This study is the first to demonstrate an essential role for syntenin in VEGF-induced angiogenesis, and VEGFR2 endocytosis and signaling in ECs.

We found that knockdown of syntenin suppressed VEGF-mediated VEGFR2 endocytosis, and VEGF-induced *in vitro* and *in vivo* angiogenesis as well as proliferation, migration, invasion, NO production, and vascular permeability of HUVECs. Moreover, downregulation of syntenin suppressed VEGF-induced VEGFR2 phosphorylation and activation of its downstream signaling molecules, as well as the expression of VEGF target genes, such as *MMP-2, VEGF, Egr1, Nurr1* and *Nur77*.

*Syntenin*-deficient mice are viable and do not exhibit any phenotypic differences [37, 38], suggesting that redundant proteins may provide compensatory signals to maintain homeostasis *in vivo*. In mice and humans, syntenin has two isoforms, syntenin (syntenin-1) and syntenin-2. Syntenin-2 shares 61% identity in amino acid sequence with syntenin-1 in mice. Although little is known about the functions of syntenin-2, its presence could compensate for the functions of syntenin in syntenin knockout mice. During development of the mouse embryo, syntenin is robustly expressed in the lung blood vessels and hepatic veins [39], suggesting a critical role in regulating endothelial cell function. It is possible that increased expression of syntenin in ECs enhances EC responses by facilitating VEGF-mediated VEGFR2 endocytosis and activation of its downstream signaling molecules.

Similar to other tyrosine kinase receptors, VEGFR2 is internalized into the cell, and this process plays an important regulatory role in VEGFR2 signaling [40–42]. In response to VEGF binding, VEGFR2 is rapidly internalized mainly in a clathrin and dynamin-dependent fashion and is trafficked to Rab5-positive vesicles [43]. VEGFR2 in endosomes was found in a complex with Nrp1, dynamin-binding protein synectin/GIPC, and myosin-VI, a reverse transport motor [43, 44]. VEGFR2 next moves from EEA1/Rab5-positive endosomes, either into recycling (Rab11-positive or Rab4-positive) endosomes or multi-vesicular bodies and late endosomes (Rab7-positive), prior to recycling or degradation, respectively [45, 46]. Initiation of VEGFR2 endocytosis requires the presence of ephrin-B2. In the absence of ephrin-B2, VEGFR2 is not endocytosed, resulting in a profound effect on its signaling [13]. Notably, the interaction of ephrin-B2 with cytoplasmic PDZ proteins through its C-terminal PDZ-bindingmotif was required for VEGFR2 endocytosis and subsequent signaling [13]. In the current study, we showed that syntenin, a tandem PDZ protein that interacts with ephrin-B2 [23, 36], regulates VEGF-induced VEGFR2 endocytosis, signaling, and angiogenesis. Syntenin knockdown impaired the interaction between VEGFR2 and ephrin-B2 and decreased VEGFR2 endocytosis and signaling, as did ephrin-B2 knockdown. This demonstrates that syntenin may function as a scaffold protein that facilitates the interaction of VEGFR2 and ephrin-B2, leading to increased VEGFR2 internalization. Notably, we found that syntenin knockdown significantly decreased the cell surface expression of ephrin-B2 and downregulated VEGFR2 signaling, both of which were rescued by MG132 treatment. Thus, syntenin appears necessary for VEGF-induced angiogenesis and VEGFR2 signaling by enhancing the cell surface expression of ephrin-B2 and thereby the interaction between VEGFR2 and ephrin-B2 to induce VEGFR2 endocytosis and signaling.

Lipid raft membrane microdomains regulate cell communication by compartmentalizing signal transduction proteins within lipid microdomains [47, 48]. Localization of VEGFR2 to lipid rafts facilitates its dimerization and endocytosis in ECs [49, 50], the steps required for VEGF-mediated signaling [9, 13]. Syntenin localized to lipid rafts in HUVECs (Supplementary Figure 5), as evidenced by colocalization of syntenin with the well-known lipid raft marker, flotillin-1, after sucrose density gradient flotation centrifugation. Thus, syntenin may function as a scaffold for the association between VEGFR2 and ephrin-B2 in lipid rafts. Loss of syntenin could decrease this interaction, resulting in a decrease in ephrin-B2-mediated VEGFR2 endocytosis and subsequent VEGFR2 signaling and angiogenesis. However, further studies are needed to determine the mechanism by which syntenin regulates the association between VEGFR2 and ephrin-B2 and VEGFR2 endocytosis in lipid rafts. The regulatory mechanism of ephrin-B2 protein stability and expression in lipid rafts is unclear. Notably, flotillin-1 regulates the level of ephrin-B2 protein in the *Xenopus* embryo [51]. Ephrin-B2 associates with flotillin-1, and loss of flotillin-1 leads to a reduction in the level of ephrin-B2 protein. This reduction is rescued by treatment with MG132 [51]. We showed here that syntenin knockdown reduced the cell surface expression of ephrin-B2 and treatment with MG132 effectively rescued this reduction. We are currently investigating whether syntenin is involved in flotillin-1-mediated stabilization of ephrin-B2 in ECs.

In summary, our study is the first demonstration that syntenin is involved in VEGF-induced angiogenesis, VEGFR2 signaling, and endocytosis by regulating ephrin-B2 function. In addition, we provide evidence that syntenin could function as a novel PDZ effector for ephrin-B2 to enhance VEGF-induced VEGFR2 endocytosis and signaling in ECs. Thus, the present work suggests that syntenin in ECs may represent a novel target for treatment of angiogenesis-related diseases, such as cancer.

## MATERIALS AND METHODS

### Cell culture

Human umbilical endothelial cells (HUVECs) were cultured on gelatin-coated plates in Medium 199 supplemented with penicillin-streptomycin (Invitrogen, Carlsbad, CA, USA), 20% heat-inactivated fetal bovine serum (FBS, Hyclone, Logan, UT, USA), heparin (Sigma-Aldrich, St Louis, MO, USA), and basic fibroblast growth factor (Millipore, Billerica, MA, USA). A549, MCF-7, MDA-MB-231, and HEK293T cells were maintained in DMEM supplemented with penicillin-streptomycin and 10% heat-inactivated FBS (Hyclone). All cells were cultured in a humidified chamber in a 5% CO_2_ atmosphere at 37°C.

### Antibodies and reagents

Anti-VEGFR2, anti-PLCγ, and anti-EEA1 antibodies were purchased from Santa Cruz Biotechnology (Santa Cruz, CA, USA). Anti-VEGFR2, anti-phospho-VEGFR2 (Y1175), anti-PECAM, anti-phospho-PLCγ (Y783), anti-phospho-Erk (T202/Y204), anti-Erk, anti-phospho-Akt (S473), and anti-Akt antibodies were purchased from Cell Signaling Technology (Danvers, MA, USA). Anti-eNOS and anti-phospho-eNOS (S1177) antibodies were purchased from BD Biosciences (San Diego, CA, USA). Anti-α-tubulin, anti-ephrin-B2, and anti-Flag antibodies were purchased from Sigma-Aldrich. Anti-syntenin antibody was purchased from Abnova (Taipei City, Taiwan). Anti-mouse secondary Alexa 488 and anti-rabbit secondary Alexa 546 antibodies were purchased from Molecular Probes (Invitrogen). Recombinant human VEGF165 and 4,5-diaminofluorescein diacetate (DAF2-DA) were purchased from Sigma-Aldrich.

### Plasmids, RNA interference, and small hairpin RNA

VEGFR2 cDNA was purchased from KRIBB (Taejon, Korea), amplified by polymerase chain reaction, digested with appropriate restriction enzymes, and cloned in-frame into the polylinker of the pCMV-HA (Clontech, Mountain View, CA). The construct was confirmed by DNA sequencing. An expression vector for VEGF (pCMV3-Flag-VEGFA) was obtained from Sino Biological Inc. (Beijing, China). Small interfering RNA (siRNA), control siRNA, and lentiviral-based small hairpin RNA (shRNA) for syntenin were purchased from Origene Technologies (Rockville, MD, USA). The sequences of siRNAs and shRNA are as follows: syntenin siRNA-A, 5′-AACCUGAAAUGUACACUAGCCCAGA-3′; syntenin siRNA-B, 5′- GAAUUCGUAGAGCAGAAAUUAAGC-3′; syntenin siRNA-C, 5′- GAAUUCGUAGAGCAGAAAUUAAGC-3′; syntenin shRNA, 5′- GTGGCTCCTGTAACTGGTAATGATGTTGG-3′. Lentiviral particles were prepared according to the manufacturer's instructions (Origene Technologies). Transfections were performed using Lipofectamine Plus reagent according to the manufacturer's instructions (Invitrogen). After transfection for 48 h, the cells were used in experiments.

### Cell proliferation assay

Cell proliferation was determined by MTT (3-(4,5-dimethylthiazol-2-yl)-2,5-diphenyl tetrazolium bromide) assay. HUVECs were seeded into 96-well plates (5 × 10^4^ cells/well) and then incubated with VEGF. After 48 h of incubation, MTT (0.5 mg/mL) was added to each well for 3 h. At the end of the incubation, the insoluble formazan products were dissolved in 200 μl dimethyl sulfoxide, and the absorbance at 540 nm was determined.

### DNA synthesis assay

Incorporation of the thymidine analog, bromodeoxyuridine (BrdU), was measured to determine the effect of syntenin on DNA synthesis using a BrdU proliferation assay kit according to the manufacturer's instructions (Millipore, Billerica, MA, USA). The assay conditions were identical to those described for the cell viability assay. HUVECs were labeled with BrdU for 4 h prior to incubation with anti-BrdU-peroxidase. The immune complex was detected by addition of trimethyl benzidine substrate.

### Cell cycle distribution analysis

HUVECs were treated with VEGF for 24 h, washed twice with PBS, trypsinized, and centrifuged. The pellets were fixed in 80% (vol/vol) ethanol for 30 min at -20°C, washed, resuspended in cold propidium iodide (PI) solution (50 μg/mL) containing RNase A (0.1 mg/mL) in PBS (pH 7.4) for 30 min in the dark. Flow cytometric analyses were performed using a FACSCalibur (Becton Dickinson, San Jose, CA, USA). Forward light scatter characteristics were used to exclude the cell debris from the analysis. CellQuest software was used to analyze the data (Becton-Dickinson).

### Immunoprecipitation and western blotting

Immunoprecipitation and Western blotting were performed as described previously [32, 33]. Briefly, cells were lysed in lysis buffer [50 mM Tris-HCl, pH 7.4, 150 mM NaCl, 1 mM EDTA, 5 mM sodium orthovanadate, 1% NP-40 and protease inhibitor cocktail (BD Biosciences, San Diego, CA, USA)], and centrifuged at 15,000 rpm for 30 min at 4°C. For immunoprecipitation, equivalent amounts of cell lysates were incubated with the appropriate antibodies followed by incubation with protein A/G agarose beads. Immunoprecipitates were extensively washed and resolved by sodium dodecyl sulfate-polyacrylamide gel electrophoresis (SDS-PAGE), transferred, and probed with the proper antibodies. The signal was detected using an enhanced chemiluminescent system (Intron, Seongnam, Korea).

### Wound healing and invasion assays

Wound healing and invasion assays were performed as previously described [27, 32, 33]. Briefly, HUVECs were seeded into 12-well plates and incubated to form a monolayer for wound healing assay. A yellow plastic pipette tip was used to make wounds across the cell monolayer. Wounded cells were incubated with VEGF for the indicated times. Phase-contrast photographs were obtained using an inverted phase-contrast microscope. For invasion assay, HUVECs (5 × 10^4^ cells/well) were seeded into the upper chambers of the filters coated with Matrigel (BD Biosciences) with 200 μL of Medium 199 (0.5% FBS). Then, 800 μL of Medium 199 (0.5% FBS) was added to the lower chamber with or without VEGF (40 ng/mL), and then cells were incubated for 24 h at 37°C. The noninvading cells were removed by cotton swab and invaded cells were fixed (Methanol 100%), stained (hematoxylin and eosin), and counted in five randomly selected microscopy fields (×100) per filter.

### Endothelial permeability assay

HUVECs were grown on gelatin-coated inserts (0.4 μm polycarbonate membrane) of Transwells (Corning Costar, NY, USA) to confluence, and then incubated with FITC (fluorescein isothiocyanate-dextran, Sigma) in the presence or absence of VEGF for the final 60 min. The amount of FITC-dextran that diffused through the endothelial monolayer into the lower chamber was measured using a microplate fluorometer (Biotek, Winooski, VT, USA).

### Tube formation assay

HUVECs were transfected with control siRNA or syntenin siRNA for 24 hr. Matrigel (130 μL) was added into wells of 48-well plates and polymerized for 30 min at 37°C. HUVECs (5 × 10^4^ cells/well) were then seeded on polymerized Matrigel and cultured for 18 h. Tubular networks in each well were photographed, and the formation of capillary-like networks was evaluated by measuring the length of capillary-like network using ImageJ software (NIH).

### *In vivo* matrigel plug assay

Animal experiments were approved by the Kangwon National University Animal Care and Ethics Committee. Matrigel plug assay was performed as described previously [46]. Briefly, 0.5 mL of growth factor-reduced Matrigel (BD Biosciences) in absence or presence of 20 ng/mL VEGF, control siRNA (2 μM, Origene Technologies), or syntenin siRNA (2 μM, Origene Technologies) was injected subcutaneously as a single plug in 6-week-old C57BL/6 mice. After 14 days, the implants were recovered, and hemoglobin content was measured using Drabkin's reagent according to the manufacturer's instruction (Sigma).

### Nitric oxide fluorometric assay

HUVECs were plated on gelatin-coated coverslips and loaded with 10 μM of DAF2-DA at 37°C for 30 min. The medium containing loading dye was removed, replaced with Medium 199, and incubated for 30 min at 37°C to ensure complete de-esterification. NO synthesis was induced by stimulation with VEGF for the indicated periods of time. Images were captured using an EVOS fluorescence microscope (AMG, Bothell, WA, USA), and fluorescence intensity was measured using the ImageJ software.

### Real-time quantitative polymerase chain reaction (qPCR)

Total RNAs were isolated using RNeasy mini kits according to the manufacturer's instructions (Qiagen, Santa Clarita, CA, USA). One μg of total RNA was used to synthesize cDNA using a Maxime RT PreMix Kit (Intron Biotechnology, Daejeon, Korea). Real-time qPCR was performed on a StepOne Real-time PCR System (Applied Biosystems). For analysis, amounts of *MMP-2, VEGF, Egr1, Nurr1, Nur77*, and *syntenin* were standardized to that of β-actin. Primers were as follows: *MMP-2*, 5′-GGCACCCATTTACACCTACA-3′ and 5′-CCAAGGTCAATGTCA GGAGAG-3′; *VEGF*, 5′-TTGCCTTGCTGCTCTACCTC-3′ and 5′-AGCTG CGCTGATA GACATCC-3′; *Egr1*, 5′-GTGCCGCTGA GTAAATGGGA-3′ and 5′-GGTCAGTGGCCTAGTG AGC-3′; *Nurr1*, 5′-GCCACGTAGTTCTGGTGGAA-3′ and 5′-GCACTCCGGGTCGGTTTAC-3′; *Nur77*, 5′-CT GGCATGAAGCGTTGTCC-3′ and 5′-GGCTCGGGGA TACTGGATACA-3′; *syntenin*, 5′-CACCATGACGATC CGTGACA-3′ and 5′-ATCAGTGAGG AGGCCGT TTC-3′; *β-actin*, 5′-ACGTTGCTATCCAGGCTGTG-3′ and 5′-GAGGGCATAC CCCTCGTAGA-3′.

### Cell surface biotinylation assay

Cell surface biotinylation assays were performed as described previously [47]. In brief, HUVECs were incubated with 0.5 mg/mL EZ-link Sulfo-NHS-SS-Biotin (Pierce, Rockford, IL, USA) in PBS on ice. After 30 min, cells were washed three times with ice-cold PBS and lysed in lysis buffer (50 mM Tris-HCl, pH 7.4, 150 mM NaCl, 1 mM EDTA, 5 mM sodium orthovanadate, 1% NP-40 and protease inhibitor cocktail). Biotinylated protein was pulled down by incubating with streptavidin-agarose beads (Pierce) at 4°C overnight. The beads were washed three times with lysis buffer and analyzed by SDS-PAGE and Western blotting.

### Biotin internalization assay

Biotin internalization assays were performed as described previously [47]. In brief, HUVECs were incubated with 0.5 mg/mL EZ-link Sulfo-NHS-SS-Biotin (Pierce) in PBS for 30 min on ice, washed three times with ice-cold PBS, and then incubated with or without VEGF for 30 min. Residual biotin at the cell surface was removed by MesNa (20 mM) in 50 mM Tris (pH 8.6) and 100 mM NaCl for 20 min on ice. MesNa was quenched with 20 mM iodoacetamide in PBS for 10min on ice. Cells were washed three times with ice-cold PBS and lysed in lysis buffer (50 mM Tris-HCl, pH 7.4, 150 mM NaCl, 1 mM EDTA, 5 mM sodium orthovanadate, 1% NP-40 and protease inhibitor cocktail). Biotinylated protein was pulled down by incubating with streptavidin-agarose beads (Pierce) at 4°C overnight. The beads were washed three times with lysis buffer and analyzed by SDS-PAGE and Western blotting.

### Confocal microscopy

HUVECs were grown on gelatin-coated coverslips in 12-well plates. HUVECs were rinsed twice in PBS, fixed in 4% paraformaldehyde in PBS for 5min, permeabilized in 0.5% Triton X-100 in PBS for 30min, and blocked in 3% BSA in PBS for 30min. The coverslips were incubated with the appropriate primary antibody. After washing three times with PBS, the coverslips were incubated with goat anti-mouse secondary Alexa 488 (for EEA1) or goat anti-rabbit secondary Alexa 546 (for VEGFR2 and p-VEGFR2) and mounted. Confocal images were acquired using a Zeiss LSM510 META NLO inverted confocal laser scanning microscope (Zeiss, Jena, Germany; Korea Basic Science Institute Chuncheon Center) equipped with an external Argon, HeNe laser and HeNe laser II. Images were captured at the colony midsection using a C-Apochromat 63× NA1.2 water immersion objective or Plan-Apochromat 100 × NA1.4 oil immersion objective (Zeiss).

### Statistical analysis

Data are expressed as means ± standard deviation. Statistical significance was assessed by two-tailed unpaired Student's *t*-test, and *P* < 0.05 was considered to indicate significance.

## SUPPLEMENTARY FIGURES



## References

[1] Carmeliet P, Jain RK (2011). Molecular mechanisms and clinical applications of angiogenesis. Nature.

[2] Carmeliet P (2003). Angiogenesis in health and disease. Nature medicine.

[3] Shibuya M, Claesson-Welsh L (2006). Signal transduction by VEGF receptors in regulation of angiogenesis and lymphangiogenesis. Experimental cell research.

[4] Rudic RD, Shesely EG, Maeda N, Smithies O, Segal SS, Sessa WC (1998). Direct evidence for the importance of endothelium-derived nitric oxide in vascular remodeling. Journal of clinical investigation.

[5] Blanes MG, Oubaha M, Rautureau Y, Gratton JP (2007). Phosphorylation of tyrosine 801 of vascular endothelial growth factor receptor-2 is necessary for Akt-dependent endothelial nitric-oxide synthase activation and nitric oxide release from endothelial cells. Journal of biological chemistry.

[6] Fulton D, Gratton JP, McCabe TJ, Fontana J, Fujio Y, Walsh K, Franke TF, Papapetropoulos A, Sessa WC (1999). Regulation of endothelium-derived nitric oxide production by the protein kinase Akt. Nature.

[7] Koch S, Tugues S, Li X, Gualandi L, Claesson-Welsh L (2011). Signal transduction by vascular endothelial growth factor receptors. Biochemical journal.

[8] Simons M, Gordon E, Claesson-Welsh L (2016). Mechanisms and regulation of endothelial VEGF receptor signalling. Nature reviews molecular cell biology.

[9] Eichmann A, Simons M (2012). VEGF signaling inside vascular endothelial cells and beyond. Current opinion in cell biology.

[10] Zhang X, Simons M (2014). Receptor tyrosine kinases endocytosis in endothelium: biology and signaling. Arteriosclerosis, thrombosis, and vascular biology.

[11] Miaczynska M, Christoforidis S, Giner A, Shevchenko A, Uttenweiler-Joseph S, Habermann B, Wilm M, Parton RG, Zerial M (2004). APPL proteins link Rab5 to nuclear signal transduction via an endosomal compartment. Cell.

[12] Nakayama M, Nakayama A, van Lessen M, Yamamoto H, Hoffmann S, Drexler HC, Itoh N, Hirose T, Breier G, Vestweber D, Cooper JA, Ohno S, Kaibuchi K, Adams RH (2013). Spatial regulation of VEGF receptor endocytosis in angiogenesis. Nature cell biology.

[13] Sawamiphak S, Seidel S, Essmann CL, Wilkinson GA, Pitulescu ME, Acker T, Acker-Palmer A (2010). Ephrin-B2 regulates VEGFR2 function in developmental and tumour angiogenesis. Nature.

[14] Wang Y, Nakayama M, Pitulescu ME, Schmidt TS, Bochenek ML, Sakakibara A, Adams S, Davy A, Deutsch U, Lüthi U, Barberis A, Benjamin LE, Mäkinen T (2010). Ephrin-B2 controls VEGF-induced angiogenesis and lymphangiogenesis. Nature.

[15] Lu Q, Sun EE, Klein RS, Flanagan JG (2001). Ephrin-B reverse signaling is mediated by a novel PDZ-RGS protein and selectively inhibits G protein-coupled chemoattraction. Cell.

[16] Lin JJ, Jiang H, Fisher PB (1996). Characterization of a novel melanoma differentiation-associated gene, mda-9, that is down-regulated during terminal cell differentiation. Molecular and Cellular Differentiation.

[17] Lin JJ, Jiang H, Fisher PB (1998). Melanoma differentiation associaed gene-9, mda-9, is a human gamma interferon responsive gene. Gene.

[18] Grootjans JJ, Zimmermann P, Reekmans G, Smets A, Degeest G, Dürr J, David G (1997). Syntenin, a PDZ protein that binds syndecan cytoplasmic domains. Proceedings of National Academy of Sciences of the United States of America.

[19] Beekman JM, Coffer PJ (2008). The ins and outs of syntenin, a multifunctional intracellular adaptor protein. Journal cell science.

[20] Sarkar D, Boukerche H, Su ZZ, Fisher PB (2008). mda-9/Syntenin: more than just a simple adapter protein when it comes to cancer metastasis. Cancer research.

[21] Zimmermann P, Meerschaert K, Reekmans G, Leenaerts I, Small JV, Vandekerckhove J, David G, Gettemans J (2002). PIP(2)-PDZ domain binding controls the association of syntenin with the plasma membrane. Molecular cell.

[22] Kang BS, Cooper DR, Devedjiev Y, Derewenda U, Derewenda ZS (2003). PDZ tandem of human syntenin: crystal structure and functional properties. Structure.

[23] Grembecka J, Cierpicki T, Devedjiev Y, Derewenda U, Kang BS, Bushweller JH, Derewenda ZS (2006). The binding of the PDZ tandem of syntenin to target proteins. Biochemistry.

[24] Baietti MF, Zhang Z, Mortier E, Melchior A, Degeest G, Geeraerts A, Ivarsson Y, Depoortere F, Coomans C, Vermeiren E, Zimmermann P, David G (2012). Syndecan-syntenin-ALIX regulates the biogenesis of exosomes. Nature cell biology.

[25] Geijsen N, Uings IJ, Pals C, Armstrong J, McKinnon M, Raaijmakers JA, Lammers JW, Koenderman L, Coffer PJ (2001). Cytokine-specific transcriptional regulation through an IL-5Ralpha interacting protein. Science.

[26] Wang LK, Pan SH, Chang YL, Hung PF, Kao SH, Wang WL, Lin CW, Yang SC, Liang CH, Wu CT, Hsiao TH, Hong TM, Yang PC (2016). MDA-9/Syntenin-Slug transcriptional complex promote epithelial-mesenchymal transition and invasion/metastasis in lung adenocarcinoma. Oncotarget.

[27] Koo TH, Lee JJ, Kim EM, Kim KW, Kim HD, Lee JH (2002). Syntenin is overexpressed and promotes cell migration in metastatic human breast and gastric cancer cell lines. Oncogene.

[28] Dasgupta S, Menezes ME, Das SK, Emdad L, Janjic A, Bhatia S, Mukhopadhyay ND, Shao C, Sarkar D, Fisher PB (2013). Novel role of MDA-9/syntenin in regulating urothelial cell proliferation by modulating EGFR signaling. Clinical cancer research.

[29] Yang Y, Hong Q, Shi P, Liu Z, Luo J, Shao Z (2013). Elevated expression of syntenin in breast cancer is correlated with lymph node metastasis and poor patient survival. Breast cancer research.

[30] Kegelman TP, Das SK, Hu B, Bacolod MD, Fuller CE, Menezes ME, Emdad L, Dasgupta S, Baldwin AS, Bruce JN, Dent P, Pellecchia M, Sarkar D, Fisher PB (2014). MDA-9/syntenin is a key regulator of glioma pathogenesis. Neuro-oncology.

[31] Boukerche H, Su ZZ, Prévot C, Sarkar D, Fisher PB (2008). mda-9/Syntenin promotes metastasis in human melanoma cells by activating c-Src. Proceedings of the National Academy of Sciences of the United States of America.

[32] Hwangbo C, Kim J, Lee JJ, Lee JH (2010). Activation of the integrin effector kinase focal adhesion kinase in cancer cells is regulated by crosstalk between protein kinase Cα and the PDZ adapter protein mda-9/Syntenin. Cancer research.

[33] Hwangbo C, Tae N, Lee S, Kim O, Park OK, Kim J, Kwon SH, Lee JH (2016). Syntenin regulates TGF-β1-induced Smad activation and the epithelial-to-mesenchymal transition by inhibiting caveolin-mediated TGF-β type I receptor internalization. Oncogene.

[34] Das SK, Bhutia SK, Azab B, Kegelman TP, Peachy L, Santhekadur PK, Dasgupta S, Dash R, Dent P, Grant S, Emdad L, Pellecchia M, Sarkar D, Fisher PB (2013). MDA-9/syntenin and IGFBP-2 promote angiogenesis in human melanoma. Cancer research.

[35] Fukumura D, Gohongi T, Kadambi A, Izumi Y, Ang J, Yun CO, Buerk DG, Huang PL, Jain RK (2001). Predominant role of endothelial nitric oxide synthase in vascular endothelial growth factor-induced angiogenesis and vascular permeability. Proceedings of the National Academy of Sciences of the United States of America.

[36] Lin D, Gish GD, Songyang Z, Pawson T (1999). The carboxyl terminus of B class ephrins constitutes a PDZ domain binding motif. Journal of biological chemistry.

[37] Tamura K, Ikutani M, Yoshida T, Tanaka-Hayashi A, Yanagibashi T, Inoue R, Nagai Y, Adachi Y, Miyawaki T, Takatsu K, Mori H (2015). Increased production of intestinal immunoglobulins in Syntenin-1-deficient mice. Immunobiology.

[38] Das SK, Guo C, Pradhan AK, Bhoopathi P, Talukdar S, Shen XN, Emdad L, Subler MA, Windle JJ, Sarkar D, Wang XY, Fisher PB (2016). Knockout of MDA-9/Syntenin (SDCBP) expression in the microenvironment dampens tumor-supporting inflammation and inhibits melanoma metastasis. Oncotarget.

[39] Jeon HY, Das SK, Dasgupta S, Emdad L, Sarkar D, Kim SH, Lee SG, Fisher PB (2013). Expression patterns of MDA-9/syntenin during development of the mouse embryo. Journal of molecular histology.

[40] Chen TT, Luque A, Lee S, Anderson SM, Segura T, Iruela-Arispe ML (2010). Anchorage of VEGF to the extracellular matrix conveys differential signaling responses to endothelial cells. Journal of cell biology.

[41] Bruns AF, Herbert SP, Odell AF, Jopling HM, Hooper NM, Zachary IC, Walker JH, Ponnambalam S (2010). Ligand-stimulated VEGFR2 signaling is regulated by co-ordinated trafficking and proteolysis. Traffic.

[42] Varsano T, Dong MG, Niesman I, Gacula H, Lou X, Ma T, Testa JR, Yates JR, Farquhar MG (2006). GIPC is recruited by APPL to peripheral TrkA endosomes and regulates TrkA trafficking and signaling. Molecular and cellular biology.

[43] Manickam V, Tiwari A, Jung JJ, Bhattacharya R, Goel A, Mukhopadhyay D, Choudhury A (2011). Regulation of vascular endothelial growth factor receptor 2 trafficking and angiogenesis by Golgi localized t-SNARE syntaxin 6. Blood.

[44] Naccache SN, Hasson T, Horowitz A (2006). Binding of internalized receptors to the PDZ domain of GIPC/synectin recruits myosin VI to endocytic vesicles. Proceedings of the National Academy of Sciences of the United States of America.

[45] Gampel A, Moss L, Jones MC, Brunton V, Norman JC, Mellor H (2006). VEGF regulates the mobilization of VEGFR2/KDR from an intracellular endothelial storage compartment. Blood.

[46] Jopling HM, Odell AF, Hooper NM, Zachary IC, Walker JH, Ponnambalam S (2009). Rab GTPase regulation of VEGFR2 trafficking and signaling in endothelial cells. Arteriosclerosis, Thrombosis, and Vascular Biology.

[47] Allen JA, Halverson-Tamboli RA, Rasenick MM (2007). Lipid raft microdomains and neurotransmitter signalling. Nature reviews neuroscience.

[48] Browman DT, Hoegg MB, Robbins SM (2007). The SPFH domain-containing proteins: more than lipid raft markers. Trends in cell biology.

[49] Ikeda S, Ushio-Fukai M, Zuo L, Tojo T, Dikalov S, Patrushev NA, Alexander RW (2005). Novel role of ARF6 in vascular endothelial growth factor-induced signaling and angiogenesis. Circulation research.

[50] Fang L, Choi SH, Baek JS, Liu C, Almazan F, Ulrich F, Wiesner P, Taleb A, Deer E, Pattison J, Torres-Vázquez J, Li AC, Miller YI (2013). Control of angiogenesis by AIBP-mediated cholesterol efflux. Nature.

[51] Ji YJ, Hwang YS, Mood K, Cho HJ, Lee HS, Winterbottom E, Cousin H, Daar IO (2014). Ephrin-B2 affects apical constriction in Xenopus embryos and is regulated by ADAM10 and flotillin-1. Nature communications.

