# Supplementary material for: Syntenin promotes VEGF-induced VEGFR2 endocytosis and angiogenesis by increasing ephrin-B2 function in endothelial cells
Source: Oncotarget. 2017 Mar 22;8(24):38886–901. doi: 10.18632/oncotarget.16452 (PMC5503580; doi:10.18632/oncotarget.16452)
Supplement: Supplementary file 1 [file oncotarget-08-38886-s001.pdf]

Syntenin promotes VEGF-induced VEGFR2 endocytosis and angiogenesis by increasing ephrin-B2 function in endothelial cells

SUPPLEMENTARY FIGURES

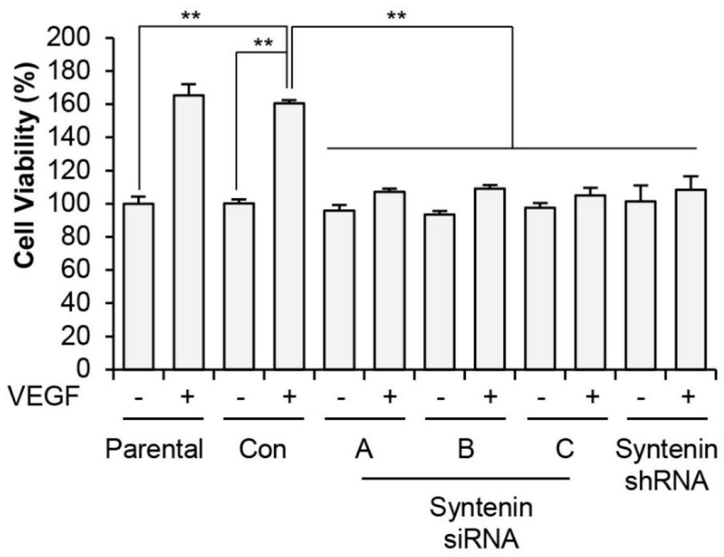

Supplementary Figure 1: Effect of three different syntenin siRNAs and a syntenin shRNA on VEGF-induced proliferation of HUVECs. HUVECs transfected with control siRNA (Con) or the indicated syntenin siRNA, or infected with lentiviral syntenin shRNA were incubated with or without VEGF (40 ng/mL) for 48 h. Cell viability was determined by MTT assay. *Columns*, mean of three independent experiments performed in triplicate; *bars*, S.D.; **, *P<0.01*.

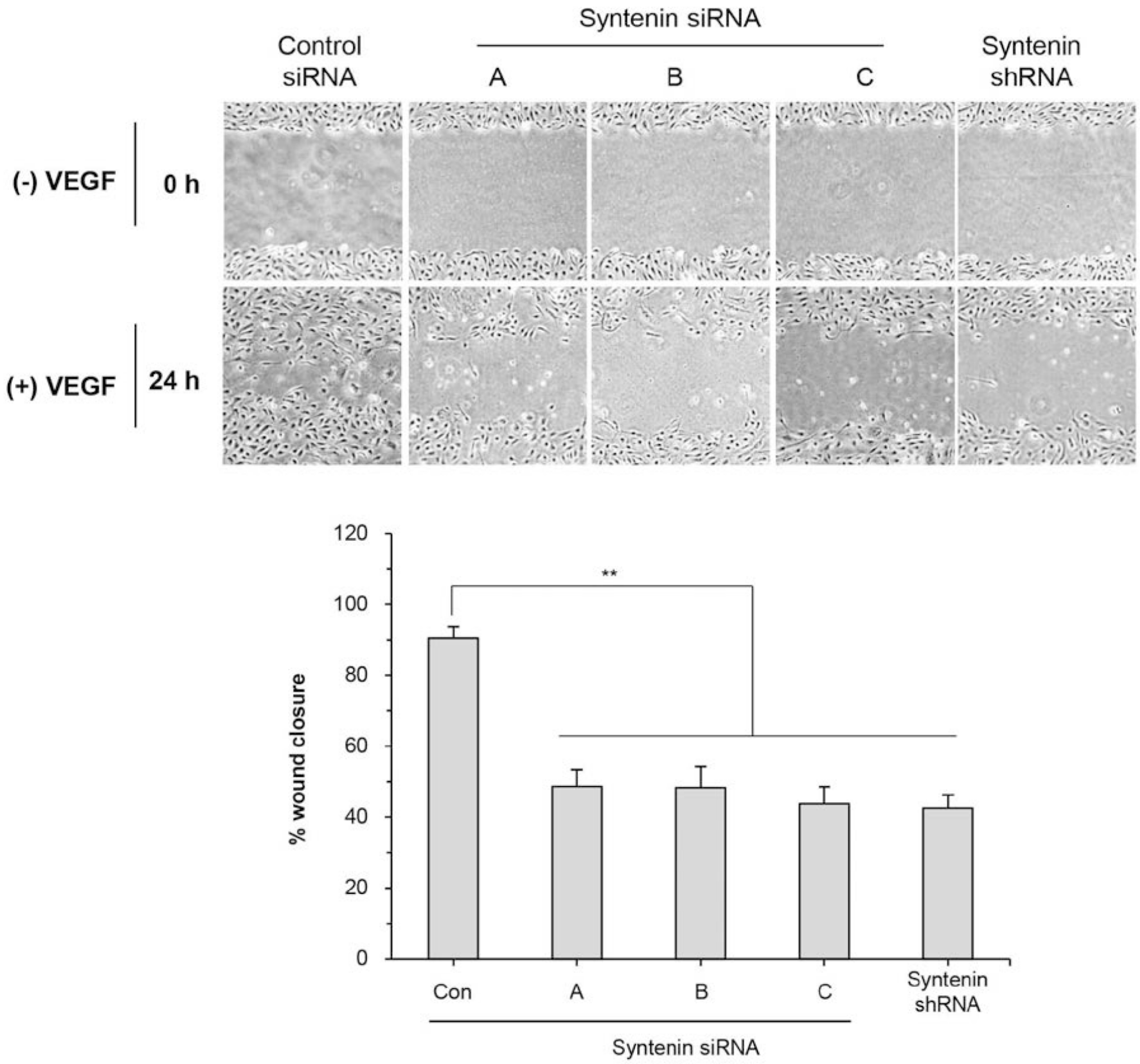

Supplementary Figure 2: Effect of three different syntenin siRNAs and a syntenin shRNA on VEGF-induced migration of HUVECs. HUVECs transfected with control siRNA (Con) or the indicated syntenin siRNA, or infected with lentiviral syntenin shRNA underwent a wound-healing assay in the presence of VEGF (40 ng/mL) for 24 h. Representative images are shown. *Columns*, mean of two independent experiments performed in triplicate; *bars*, S.D.; **, $P<0.01$.

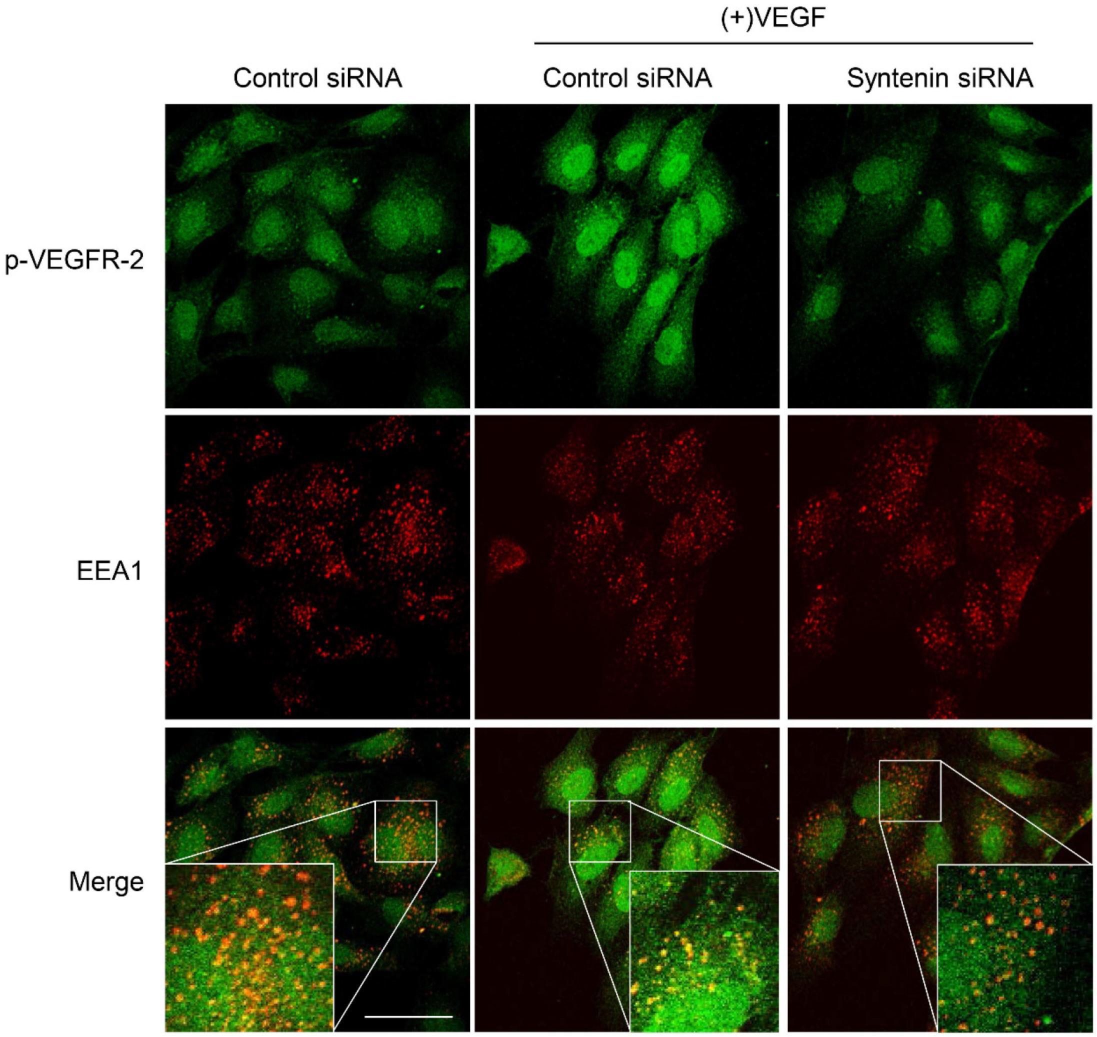

Supplementary Figure 3: Effect of syntenin downregulation on the VEGF-induced colocalization of phospho-VEGFR2 with EEA1. HUVECs transfected with control siRNA or syntenin siRNA were incubated with VEGF (40 ng/mL) for 15 min, and then immunostained with phospho-VEGFR2 or EEA1 antibody. Representative images of immunostaining results are shown. *Bar* = 50 µm.

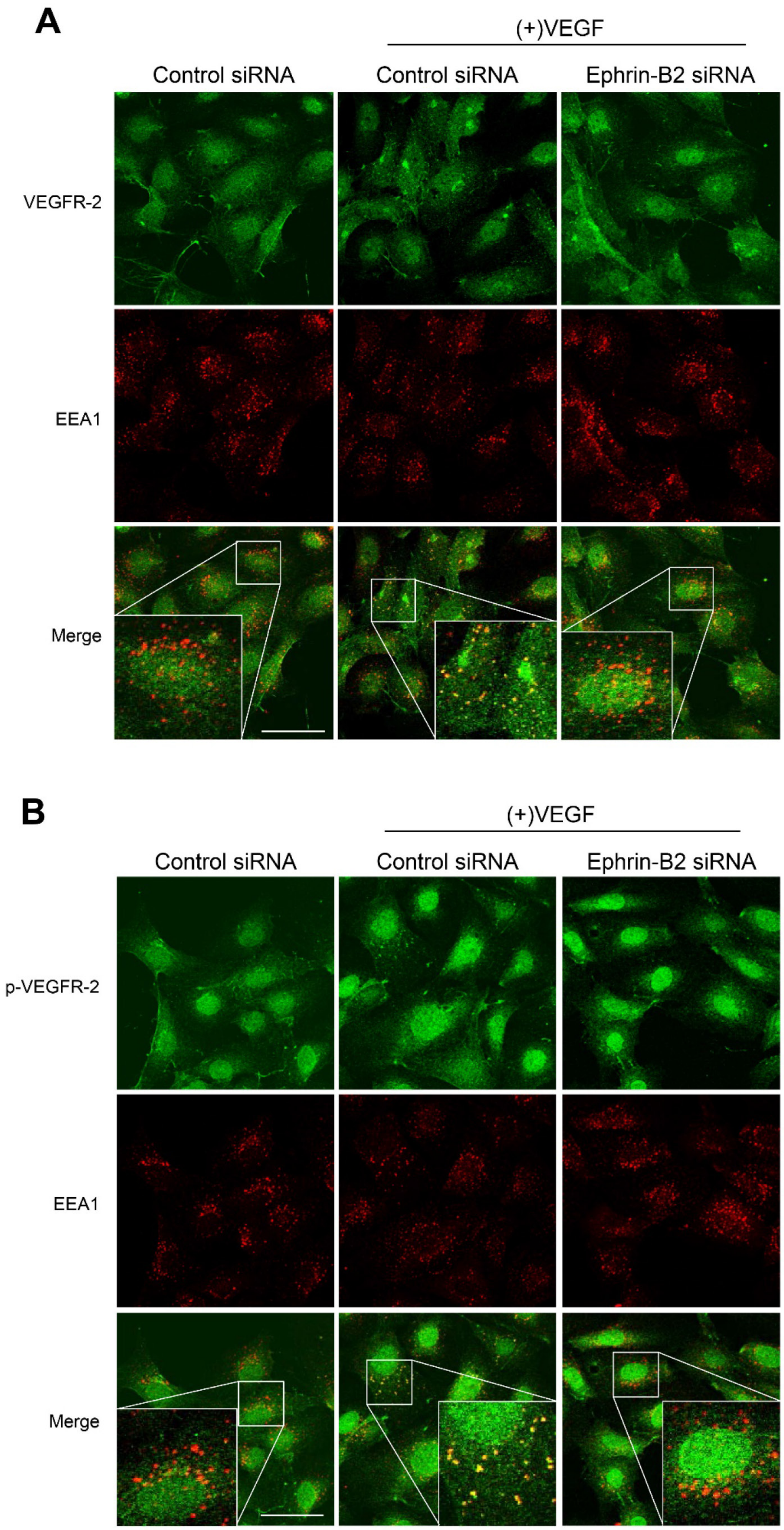

Supplementary Figure 4: Effect of ephrin-B2 downregulation on the VEGF-induced internalization of VEGFR2. (A) HUVECs transfected with control siRNA or ephrin-B2 siRNA were incubated with VEGF (40 ng/ml) for 15 min, and then immunostained with VEGFR2 or EEA1 antibody. Representative images of immunostaining results are shown. Bar = 50 µm. **(B)** HUVECs transfected with control siRNA or ephrin-B2 siRNA were incubated with VEGF (40 ng/ml) for 15 min, and then immunostained with phospho-VEGFR2 or EEA1 antibody. Representative images of immunostaining results are shown. *Bar* = 50 µm.

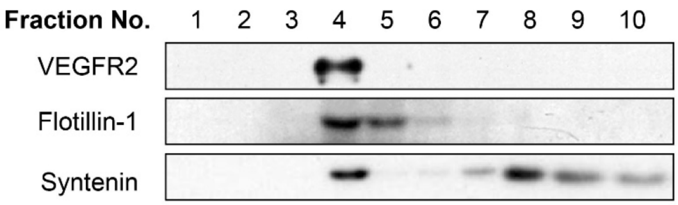

Supplementary Figure 5: Syntenin and VEGFR2 localize to lipid rafts in HUVECs. HUVECs were harvested for lipid raft purification using sucrose density gradient centrifugation. Fraction aliquots were immunoblotted with the indicated antibodies. Flotillin-1 was used as a well-known lipid raft marker.
